# Bridging the gap: umIT makes complex imaging data accessible to scientists of all backgrounds

**DOI:** 10.1117/1.NPh.12.S1.S14616

**Published:** 2025-08-22

**Authors:** Bruno Oliveira Ferreira de Souza, Montana Samantzis, Catherine Albert, Samuel Belanger, Jean-Francois Bouchard, Matilde Balbi, Matthieu P. Vanni

**Affiliations:** a Labeo Technologie Inc., Montréal, Québec, Canada; b The University of Queensland, Queensland Brain Institute, Brisbane, Queensland, Australia; c Université de Montréal, École d’Optométrie, Montréal, Québec, Canada; d Université de Montréal, Centre Interdisciplinaire de Recherche sur le Cerveau et l’Apprentissage (CIRCA), Montréal, Québec, Canada

**Keywords:** toolbox, mesoscale imaging, calcium imaging, big data, MATLAB

## Abstract

**Significance:**

In recent years, numerous open-source tools have been developed to facilitate data analysis in neuroscience, significantly encouraging the use of high-throughput approaches and promoting standardizing methods. Tools for macroscopic mapping (e.g., magnetic resonance imaging, electroencephalogram) and microscopic techniques (e.g., multi-electrode electrophysiology, calcium imaging) are now widely available.

**Aim:**

However, at the intermediate spatial level, the mesoscopic scale, there is a lack of equivalent open-source resources even though this scale is crucial for understanding the function of cortical maps. Optical techniques such as calcium imaging are well suited to investigate this scale, enabling measurements of cortical responses and functional connectivity. Yet, analyzing complex, multiparameter datasets remains challenging. Existing toolboxes are restricted in handling the complexity of such data, limiting their utility for mesoscale studies.

**Approach:**

To address these challenges, we propose the Universal Mesoscale Imaging Toolbox (umIT), an open-source MATLAB-based platform developed to analyze large-scale imaging datasets.

**Results:**

umIT supports a comprehensive, streamlined workflow accessible via both a graphical user interface and command-line interface, eliminating the need for third-party software.

**Conclusions:**

This toolbox aims to make mesoscale imaging more accessible and transparent, facilitating robust comparisons across regions, groups, and time points (longitudinal studies). Importantly, umIT was also designed to facilitate intuitive interaction with mesoscale data, an aspect that may be particularly valuable for trainees who are just beginning to work with wide-field optical imaging.

## 1 Introduction

In recent years, the development of open-source tools and software shared within the neuroscience community has facilitated the analysis of various experimental modalities. The adoption of these tools had a significant impact on the development of cutting-edge, high-throughput approaches while also helping to standardize methods, thereby making procedures more transparent and comparable. For instance, in macroscopic mapping, tools such as Brainstorm or statistical parametric mapping^1^ have streamlined the analysis of magnetic resonance imaging and electroencephalography. At the microscopic level, platforms such as Open Ephys and Spike sorting Kilosort for multi-electrode electrophysiology as well as Fiji^2^^–^^4^ or CellPose^5^ for calcium imaging data from multiphoton microscopy have maximized the potential of these techniques. However, although both macroscopic and microscopic scales benefit from a wide range of resources, the intermediate scale, also known as the “mesoscale,” lacks open-source tools that allow the same level of advancement.

The mesoscale is critical for understanding information processing and the spatial organization of the cortex. At this level, cortical maps, such as somatotopy in the somatosensory and motor cortex or retinotopy, orientation maps, and ocular dominance columns in the visual cortex, can be extensively described.^6^^–^^8^ Common approaches for exploring mesoscale dynamics are primarily optical, including intrinsic signal imaging,^9^ voltage-sensitive dye imaging,^10^ and more recently, widefield calcium imaging.^11^ These approaches allow two-dimensional imaging of cortical responses to sensory or cortical stimulation by measuring spatial changes in reflectance or fluorescence of calcium indicators such as variants of GCaMP.^12^ Furthermore, by recording spontaneous activity during rest, functional connectivity can also be assessed using methods, including Pearson correlation between two regions.^9^^,^^13^ Connectivity maps can also be generated by measuring the correlation between a reference point and each of the other points in the cortex (seed pixel correlation). Another advantage of these approaches is their ease of use in a multimodal context on awake animals performing behavioral and motor tasks.^14^^,^^15^ In addition, they enable the study of brain functions longitudinally or as a function of brain states.^16^^,^^17^

For the analysis of mesoscale imaging data, many research teams still rely on custom-built pipelines to measure differences in cortical maps across experimental conditions. However, it becomes increasingly complex, as seen in longitudinal studies, when parameters multiply and involve several time points. Although some toolboxes have been developed to address these complexities, they are often restricted in their ability to manage complex datasets with multiple parameters of modalities simultaneously.^18^^–^^22^ These limitations may have hindered their broader adoption to date. Therefore, there remains a clear need for the development of more toolboxes capable of comparing functional responses across many regions, from multiple animal groups, over time.

To address these challenges, we introduce Universal Mesoscale Imaging Toolbox (umIT), an open-source MATLAB-based solution designed to meet the unique demands of mesoscale imaging. umIT provides an analysis pipeline that supports large imaging datasets, offering flexibility through a graphic user interface (GUI) or command-line interface. It was designed to enable complete analysis workflows from start to end, in a controlled manner, without the need for any third-party software. The adoption of umIT aims to make mesoscale imaging more accessible, facilitating comparison across cortical regions, groups, and timepoints while integrating multiple imaging modalities.

## 2 Description of the Toolbox

### 2.1 Overview of umIToolbox

This paper introduces the main features of the umIT toolbox. All resources, including detailed tutorials, are accessible on the wiki page: https://labeotech.github.io/Umit/, and the source code is available on GitHub: https://github.com/LabeoTech/Umit

umIT is dedicated to the processing, visualization, and analysis of large mesoscale imaging datasets. It works through two main applications: DataViewer and umIToolbox. DataViewer is tailored for the visualization and processing of single acquisitions.

**Fig. 1:**
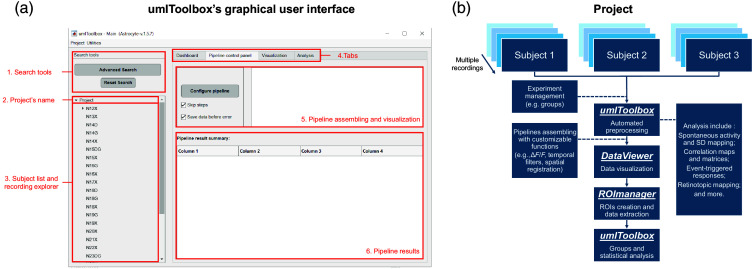
General organization of the umIToolbox. (a) Single Snapshot of umIT’s GUI for the pipeline control panel tab. (b) Schematic of umIT’s hierarchy as a user is managing a longitudinal project containing many subjects with multiple acquisitions each, of different modalities, recorded over a long period of time.

The umIToolbox is written in MATLAB and provides a structured environment where the user can manage the data, automate processing pipelines, and visualize results in a single graphical interface. During development, significant efforts went into designing the toolbox to optimally manage large datasets involving varied measurements, across multiple groups of subjects and time points, allowing for comparisons and statistical analyses. Considerable attention was simultaneously given to ensure usability, making this toolbox also accessible and easy to handle for users with limited MATLAB knowledge or data analysis experience. Although all functions can be accessed using command lines or scripts, the toolbox was primarily developed with an intuitive and versatile GUI [Fig. 1(a)].

This tool automates the analysis of large datasets. For example, it can compare changes in functional connectivity among different cortical regions in mice who suffered a brain injury and control over time, using calcium and hemodynamic signals (involving multiple acquisitions, two groups of several subjects, and dual modalities). Similarly, it can measure responses to sensory stimuli between mice across several treatment groups and time points.

A typical imaging project in umIToolbox consists of one or more subject groups (e.g., mice) that undergo one or more acquisition (i.e., recordings) sessions over time [Fig. 1(b)] that can include different modalities (e.g., behavioral responses and sensory stimulation) associated with the imaging data. This toolbox facilitates fully exploiting imaging datasets by replacing other software needed from initial data management to generating final figures. The different functionalities of this toolbox, which will be described below, thus include (1) data management, (2) preprocessing, (3) analysis, (4) visualization of cortical maps, and (5) data quantification of regions of interest.

### 2.2 Data Management

The toolbox offers a GUI to organize multiple recordings to efficiently process them. By default, the raw data of each recording consists of a series of binary files located in a folder, as generated by the Light Track IOS200 imaging systems used in our laboratories (LabeoTech Inc.), but it also accepts other file formats, including TIF files converted from different imaging systems. When creating a typical imaging project, information on the data organization, such as the raw and analyzed data location directions, is automatically stored in a MATLAB file named after the project. This file also contains each subject identity, recordings, and acquisition modality, which are then indexed to filter and organize them thanks to one function (“protocolFcn.m”). The same function, which can be adapted based on a project needs, can then allow users to update the whole project periodically by adding or removing raw data files for existing and new subjects without impinging on the remaining dataset. Subjects can also be bundled into groups, along with subject information such as sex, strain, or age, to facilitate analysis, and all information can be edited if needed.

#### 2.2.1 Main files

The raw data (e.g., bin or TIF files) are read using one of the available data import functions (https://labeotech.github.io/Umit/documentation/userDocs/fcns/dataImport_index.html). For TIF files, the meta data (frame rate, exposure time, illumination color, etc.) are stored in a .JSON file. Once imported, the imaging data are stored as binary files (.dat) with an associated .mat file containing the recording’s metadata (e.g., frame size, rate, and number of frames). Each .dat file contains the image time series from a single recording channel. In cases of interleaved illumination, each channel (red, green, fluorescence, etc.) is saved in a separate file.

#### 2.2.2 Event files

Event files can be stored alongside imaging files to enable specific analysis operations such as event-triggered responses, which allows measurement and comparison of sensory responses to different stimuli. Events are stored in an events.mat file containing the events’ timestamps (in seconds), state (onset/offset), event indices, and a list of event names. This file is then used to split the image time series in the data processing to generate event-triggered average responses (e.g., block averaging based on the trigger file).

### 2.3 Preprocessing

Once organized, the data can be preprocessed and analyzed by assembling pipelines made out of serial, customizable functions. The processing pipelines can then be automatically executed across all folders within a given project. The available functions include common preprocessing routines used in mesoscopic imaging such as hemodynamic correction,^23^^,^^24^ global activity regression,^25^^–^^28^ temporal filters,^29^ normalization (ΔF/F), splitting, and classifying data into events and spatial registration.^18^ Custom-made functions can be integrated into the software given that they use the toolbox syntax (detailed explanation can be found at https://labeotech.github.io/Umit/documentation/userDocs/other/how-to-create-custom-functions.html). For example, one could create a custom function to split the data into separate frequencies to allow computing the power spectra of the signal or the separation of the signal into frequency bands.

Hemodynamic correction is based on parallel measurements from multiple imaging channels to capture intrinsic signals alongside fluorescence. This approach allows for the removal of fluorescence signals from hemodynamic artifacts. Thus, a series of reflectance images in the red, orange, and green wavelengths can be collected using a second camera or a sequential illumination device (strobing). These reflectance signals can then be used to regress out artifacts from the fluorescence channel^23^ using a pixel-wise linear regression of the fluorescence signal onto the reflectance signals. This approach was chosen because of its ease of use as it requires very few input parameters. However, alternative methods, such as the ratiometric approach, can also be applied. Changes in HbO and HbR concentrations could also be estimated from reflectance at different wavelengths using the modified Beer–Lambert law and the specific absorption spectra of each hemoglobin species.^30^ The flexibility to modify spectral parameters, including filter/illumination settings and camera spectral profiles, enables the method to be applied across a range of imaging systems.

Many other computations can also be applied to enhance the significance of the signal. For example, the global activity regression can be used to calculate the average common signal across all pixels and remove it. This type of procedure is very useful to increase the contrast of functional maps but makes the interpretation of measurements more complex. Temporal filters allow for the removal of temporal components such as high and/or low-frequency signals, which can also compromise the purity of the analysis. Finally, normalization of the signal by the mean fluorescence (ΔF/F) is a classic procedure in functional fluorescent imaging to measure the responses in % of variation and to substitute the level of expression of the signal on the cortical surface.

### 2.4 Analysis

Cleaned, preprocessed data can now be analyzed to evaluate functional responses or connectivity during spontaneous activity by creating correlation maps and matrices.^9^^,^^13^^,^^29^ The Pearson correlation coefficients are therefore calculated between the spontaneous activity of a reference point, the seed, and other reference points to generate seed pixel correlation maps depicting the connectivity of a region with all the others. In parallel, creating connectivity matrices where the correlation coefficients are calculated among several chosen pairs of ROIs is also doable. This approach does require objectively defining the regions of interest (ROI), which will be described in Sec. 2.6. For both correlation maps and matrices, a Fisher z-transformation can be applied to Pearson’s correlation values. This transformation is frequently used as a processing step to approximate the data to a normal distribution before statistical analysis. Spontaneous activity changes can moreover be estimated by measuring the standard deviation of the signal for each pixel (SD) at each pixel as more fluctuations will be associated with more variability around the average.

Event-triggered responses (e.g., for sensory stimuli) can also be investigated as times and categories of events, loaded from .csv or .txt files, which identify the temporal location of specific responses within a recording. Individual sequences of normalized responses preceding and following each event (trial) can then be grouped and averaged.

Although subjects are typically positioned consistently during imaging sessions, the toolbox includes a versatile spatial registration tool to register data both manually and automatically. In both procedures, the user creates an imaging frame from a given recording (normally the first one) as a reference. To perform a manual registration, the user first interactively selects identical landmarks in both the reference frame and the imaging frame from the unregistered recording. Next, a local approximation algorithm registers the frames by generating and applying a geometric transformation to the target data after translating, rotating, and scaling the frames to match. This approach was chosen because it is generally quite easy to identify characteristic features in blood vessel patterns between multiple recordings. If done automatically, the registration is performed using MATLAB’s imregister function with optimization hyperparameters that were selected to maximize a mutual information criterion. Inter-subject registration, which only applies translation or scaling to the registration, can be used to create averaged maps per group by considering selected reference points (e.g., Bregma or ROIs) and the pixel ratio of each recording. Transformation matrices (tform) used in the automatic or manual registration are then saved in the file “tform_info.mat” in the save folder.

### 2.5 Visualization of Cortical Maps

**Fig. 2:**
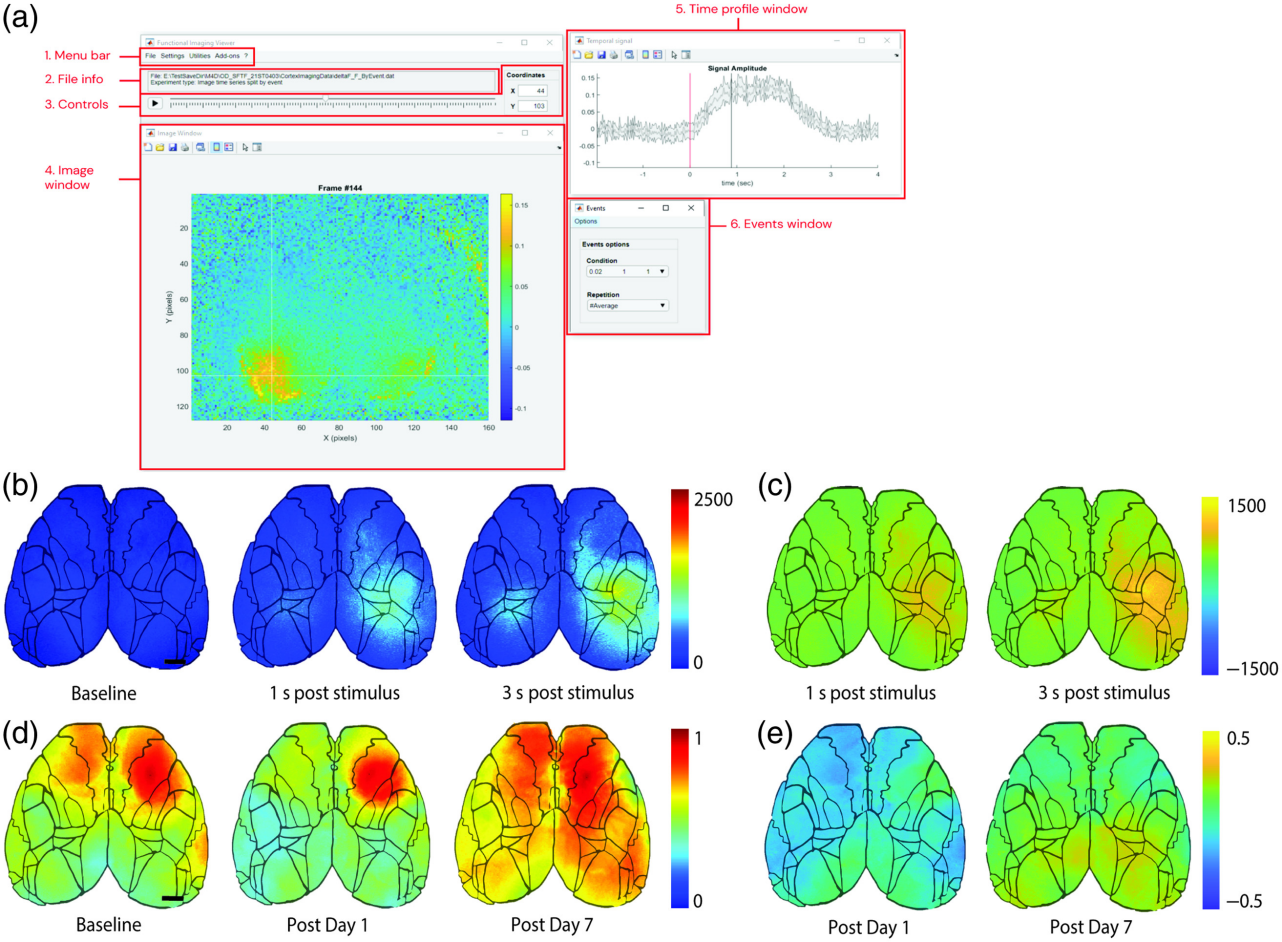
Cortical maps. (a) Representative image of umIT’s GUI when analyzing event-triggered responses. (b) Average maps (n=4) of peak response amplitude following a whisker stimulus to the left-hand side of the face. (c) Same maps as in panel (b) but subtracted from the baseline. (d) Average seed pixel correlation maps for a seed in M1 before and after a cortical lesion in V1. (e) Same maps as in panel (d) but subtracted from the baseline.

Once the data have been analyzed, the toolbox supports the visualization of cortical maps for event-triggered responses and seed pixel correlation. For event-triggered responses, the GUI provides a clear user interface to visualize changes across the recording period [Fig. 2(a)]. Montages of the calcium signals preceding and following events can be averaged across subjects within a group [Fig. 2(b)]. It is also possible to calculate different metrics from the temporal profiles of each pixel, such as the peak amplitude and latency, onset latency (delay for the signal to cross a threshold, set as a multiple of the standard deviation of the pre-event signal) or area under the curve (AUC) amplitude, and presenting them based on the experiment times (e.g., longitudinal measurements), modalities (e.g., intensity or frequency), subjects or groups. Temporal dynamics can also be displayed as subtraction maps, showing changes relative to a baseline or reference map [Fig. 2(c)]. The ability to incorporate custom functions into the pipeline could also allow the user to include more sophisticated analysis approaches such as the application of a linear GLM model.^21^ Spontaneous activity can be similarly visualized by calculating the SD across pixels for a specific time course. To improve the interpretation and avoid incorporating erroneous data, an overlay of ROIs (e.g., Mouse Allen Brain Atlas, drawn manually or thresholded) can be added on the maps as well as a logical mask hiding regions outside the visible cortex. Next, the seed pixel correlation maps can also be orderly displayed based on acquisition times, modalities, subjects, or groups with or without the application of a subtraction as previously described for the event-triggered responses [Figs. 2(d) and 2(e)].

### 2.6 Quantification in Regions of Interest

Even if they are quantitative, the maps previously described do not allow easy comparisons between regions. The toolbox therefore allows one to measure the activity in specific regions of the cortex—the regions of interest (ROIs)—which can be edited in different ways using the dedicated app ROImanager. The users can manually draw ROIs as single points, circles, or polygons use pre-established ROIs such as those defined by the Mouse Allen Brain Atlas^31^ or use the image’s pixel values to select a region with values above or below a determined threshold. This last feature is particularly useful to delimit regions based on the signal amplitude. ROIs can also be modified and combined. For example, ROIs can be cropped, based on logical masks corresponding to the visible cortical field of view, and merged. Finally, they can be saved for reuse in subsequent studies or shared with collaborators.

After creating and saving ROIs, users can extract and aggregate the data from individual ROIs across multiple recordings that were imported, preprocessed, and separated into experimental groups. Visualization of grouped data can be performed in two main ways: (1) to plot evoked responses (or spontaneous activity) as a function of time in ROIs or (2) to plot connectivity matrices to visualize the correlation of signals between ROIs.

**Fig. 3:**
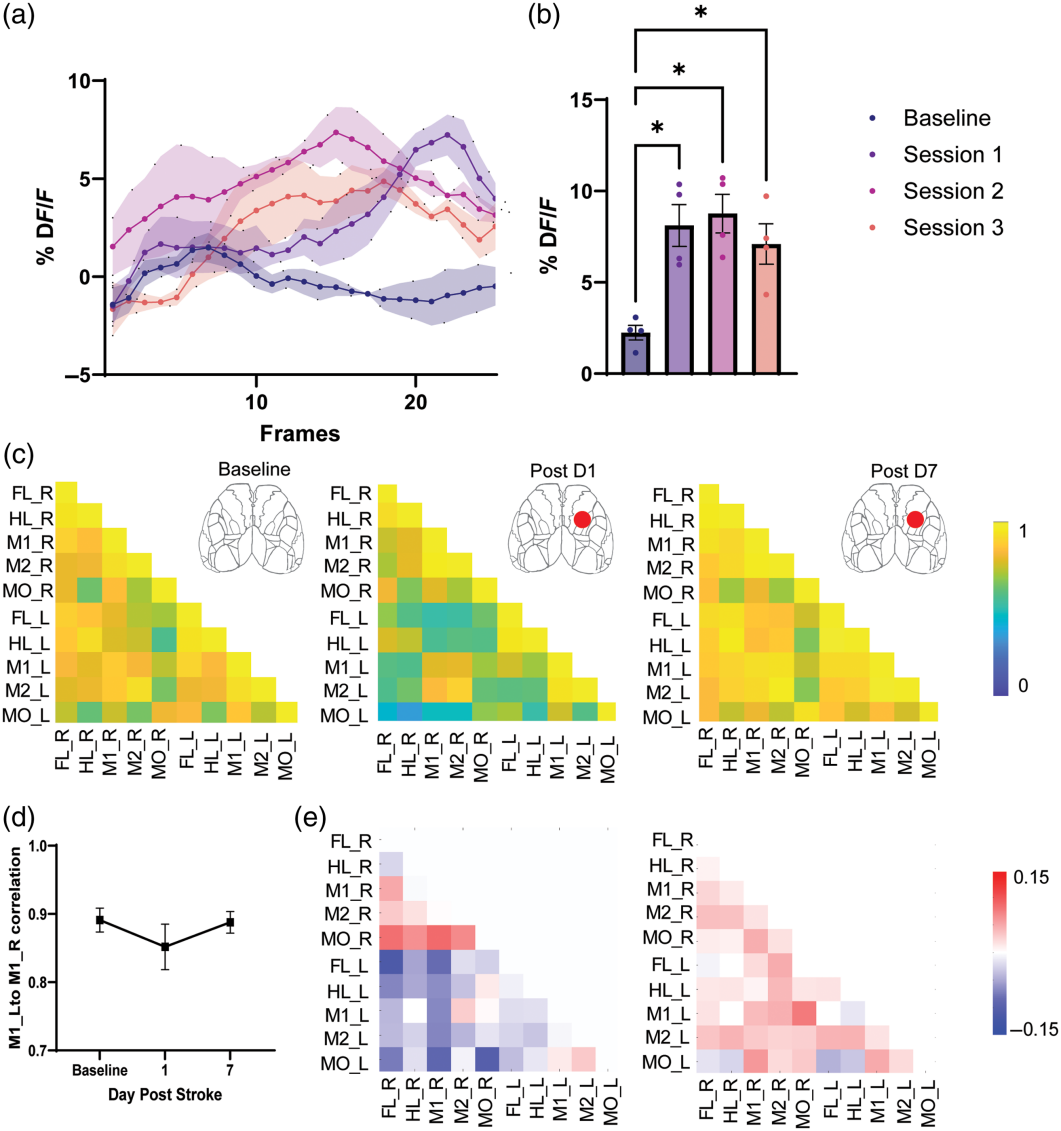
Seed pixel correlation matrices. (a) Average profiles of responses (n=4, shaded area: ±SEM) in the ROI located on the barrel cortex (BC) following a contralateral whisker stimulus and for four different acquisitions (baseline and during three stimulation sessions). (b) Average peak amplitude of response in BC over different stimulus sessions. (c) Average correlation matrices for different acquisitions before and after a cortical lesion in the right area M1. (d) Average correlation between homotopic areas M1 before and after a cortical lesion in the right area M1. (e) Same matrices as in panel (c) but subtracted from the baseline.

The evoked response plots display the profile of the average signals before and after the event of different ROIs, acquisitions (e.g., longitudinal measurements), modalities (e.g., stimulus intensities), subjects, or groups, depending on the user’s chosen parameters [Fig. 3(a)]. Averaged signals as well as their error bars (e.g., SD or SEM) can be added to account for the variability between trials of the same recording or among different subjects of a group. Instead of plotting the profile around the event, one could choose to plot metrics extracted from the temporal profiles, such as peak amplitude and latency, and compare the metrics of different ROIs, acquisitions, modalities, subjects, or groups [Fig. 3(b)]. When correlation matrices (or averages) are generated, it is then possible to display them according to subjects, groups, acquisitions, or modalities [Fig. 3(c)]. Another way to visualize correlation matrices is to subtract each matrix from a reference matrix to better highlight the changes in connections over time after a manipulation [e.g., a lesion, Fig. 3(e)]. As such, this procedure allows for tracking the evolution of the measures but does not constitute a statistical approach. Therefore, we have also included in the toolbox a set of simple models that enable statistical testing adapted to certain experimental scenarios. The statistical tests available in the toolbox accommodate common experimental designs, such as single-group comparisons before and after treatment, control versus treatment groups, and control versus treatment over time. The toolbox automatically selects an appropriate test based on how the data are structured. For instance, if the dataset consists of two groups of mice (control versus treatment) with a single measurement per group, an independent two-sample t-test is applied. Nonparametric versions of the tests are used if the data are not normally distributed or, in case of ANOVA, fail to meet the homoscedastic criterion. Finally, the user can plot the evolution of a connection (a point in a matrix) between subjects, groups, acquisitions, or modalities [Fig. 3(d)].

To incorporate more specific features into the pipeline, users can create their own custom functions and add them to the toolbox. For example, one could implement a PCA/ICA algorithm within a custom-made function as mentioned previously. Then, it could be possible to import the resulting ROIs into the ROI manager for inspection and manual refinement. In addition to the ability to create custom functions, imaging data can also be exported as .TIF files, whereas grouped data can be exported as .CSV or .MAT files. This allows users to continue their analyses using other software platforms if needed.

In conclusion, this toolbox can handle mesoscopic imaging data at all stages of the analysis, from management, preprocessing, analysis to visualization. The features offered in this version of the toolbox are obviously not exhaustive but cover a large part of the classic needs in the field. As everything is operated in MATLAB, all the processed data, whether final or intermediate, are accessible in the workspace. It is then easy for the user to develop subsequent analyses using MATLAB scripts or to export the data to another software.

## 3 Discussion

### 3.1 How to Deal with Big Data in the Field of Mesoscopic Imaging

This toolbox addresses a significant gap in the field of mesoscopic imaging: the challenge of managing and analyzing complex, large-scale datasets. Developing scripts for the analysis of a single acquisition is something rather easy and several Open-Source tools already exist (see below). However, having a tool able to combine several recordings, from several measurement points in time and from several subjects belonging to different subgroups, is much more complex to design, especially for teams that do not have access to qualified personnel to develop these computational features. Until now, in contrast to single acquisition needs, no tool was available to deal with large mesoscopic datasets. Our approach of developing a tool that the community could use freely, and that evolves, therefore made sense.

This toolbox was therefore designed, from the beginning, to deal with the complexity of large datasets to easily exploit them from beginning to the end, without having to use other third-party software. To facilitate its use, emphasis was put on the graphical user interface (GUI) and the pipeline concept, which makes it easier to follow the sequence of operations performed. To combine multiple measurements, we have dedicated a significant part of the development to providing various solutions for the registration of recordings. As the quantitative aspect was very important, we have also incorporated functionalities to manage measurements of regions of interest (ROI). These can be imported, edited, merged, or created. Finally, the ability to incorporate different imaging channels makes it possible to carry out hemodynamic measurements^30^ or to apply a hemodynamic correction to fluorescent signals.^23^

### 3.2 Examples of Application

Sharing this tool should notably promote the use of mesoscopic imaging in many subsequent studies requiring longitudinal measurements of resting state connectivity and evoked responses. It is particularly relevant for studies investigating plasticity, development, or the impact of experimental interventions on the cortical circuits between groups of mice over time.^26^^,^^32^^–^^34^ This suitable tool could be associated with high-throughput automated imaging devices made for mice.^35^^–^^37^ These strategies generate large quantities of small imaging sequences, coming from several mice, which would be almost impossible to process manually. Finally, although this toolbox was primarily developed for calcium imaging applications in mice, it can be effectively used for other imaging modalities, such as intrinsic signals or voltage imaging^38^ or in other species such as primates,^39^ tree shrew,^40^^,^^41^ or cats.^42^^,^^43^ Its use in *in vitro* applications, for example, in brain slices, could also be considered.^44^

### 3.3 How Does umIT Supplement Available Toolboxes?

Among online toolboxes, Mouse_WOI combines the most common features with umIT^21^ because this open-source MATLAB toolbox can also exploit large datasets to explore functional connectivity as well as evoked responses. It also has many features superior to umIT when it comes to analyzing functional connectivity as well as statistical evaluation of maps. The main difference lies in managing complex graphs that combine averaged data of multiple groups, ROIs, and acquisitions over time. An experienced programmer will most likely prefer the freedom offered with writing their own scripts, whereas a newcomer, with little to no background in coding, might find it overwhelming and could therefore appreciate the more streamlined approach offered by umIT to generate figures.

Other initiatives have also shared toolboxes that allow fairly advanced investigations on functional connectivity in mice using BioImage Suite, an Open medical imaging analysis software package.^19^ Similar to umIT and Mouse_WOI, this workflow, “BIS-MID,” offers for very comprehensive data preprocessing as well as detailed comparisons of connectivity maps between groups of mice. It therefore focuses on comparing resting-state connectivity mapping across different conditions. Similar to the mesoscale brain explorer (MBE), another Open Toolbox developed in Python that also has the ability to combine multiple recordings after registration and do elaborate processing,^18^ they do not include functionality for managing evoked data or integrating other modalities. Moreover, MBE cannot establish comparisons among different subject groups or manage multiple imaging channels for hemodynamic correction.

Several other mesoscopic imaging data analysis toolboxes have also been shared, but most of them can only analyze one acquisition at a time and are therefore more compatible with one-time experiences as opposed to longitudinal projects that come with large imaging datasets combining repeated recordings on multiple subjects. Among them, VobiOne is a toolbox integrated with BrainVISA, an open-source software platform dedicated to the analysis of neuroimaging data.^20^ Its aim was to generate evoked responses in different conditions (e.g., contrast) along with comparing and testing hypotheses on how to denoise and preprocess data. It is therefore equipped with a large panel of functionalities such as general linear models (GLM) or spectral analysis that the user can benchmark to evaluate the impact. BrainVISA is indeed equipped with a data management strategy relying on the use of a database to index data, but it is not clear if VobiOne can handle data from different acquisitions and subjects. It also does not have any functionality related to the analysis of resting-state connectivity.

In the end, choosing an appropriate toolbox falls into the hands of the users and widely depends on the goal of their project. Suitable especially for longitudinal projects, umIT was created to handle large imaging datasets involving multiple acquisitions over time, from different modalities and from multiple subjects of different groups, to explore both functional connectivity and evoked responses within one accessible application. It allows the user, whether new to programming or not, to interactively manage its data along with the pipeline of procedures and to update them at will. Elaborate automatic registration features are also available to allow averaging of multiple animals and acquisitions to easily present consensus maps from different subjects or time points. Due to the versatile ROI management features included in the toolbox, another notable aspect is the ability to generate interactive graphs in an intuitive way with the GUI, thus making it feasible to compare responses and connections between ROIs of different groups or subjects over time.

### 3.4 Limits and Future Developments

When designing this toolbox, great care was taken to ensure that as many steps as possible could be semi-automated, with possible supervision through the pipeline. However, many steps require active user participation, such as identifying key features (e.g., bregma and lambda), delineating the visible cortex of the recording to apply a logical mask, or organizing the dataset when opening and updating the project. These manual steps can slow down the analysis process. In the future, several of these manual steps could be replaced by automated approaches, thanks to the development of AI approaches. For example, using machine learning, it is now possible to place atlases in a fully automated way by training networks to recognize features or the spatial structure of cortical activity.^22^

So far, the toolbox is limited to exploiting evoked response and functional connectivity through correlation measurements, which covers a large part of the usual needs in the field. In the future, the adoption of this toolbox by new users will help identify specific, recurring needs, thereby enabling the addition of new functionalities. This could include clustering and graph analysis tools,^45^^,^^46^ spectral analysis, and data modeling options such as estimating contrast sensitivity by fitting response profiles with the Naka–Rushton equation. ^47^

Although the toolbox allows elaborate quantifications, it currently offers basic support for statistical analyses that will be further advanced in upcoming versions. The metrics generated are, however, fully accessible in the MATLAB workspace and can be easily manipulated or exported to other specific applications. We will also take advantage of the adoption of this toolbox by several teams to identify consensus approaches for the statistical tests to apply, which we will integrate using MATLAB’s Statistics and Machine Learning Toolbox.

Although MATLAB is widely used in the field of neuroscience and neuroimaging, not all universities have access to it, which could limit the development of the toolbox. Thus, we will consider developing parallel versions of the toolbox both in MATLAB and Python in the future to broaden adoption in a larger user community.

Another challenge yields in the standardization of approaches to better compare published results. This is at the heart of several initiatives such as the International Brain Laboratory to normalize the behavioral procedures.^48^ Distributing an analysis toolbox where everyone could share and use the same tools, which are accessible through the pipeline, would improve transparency and standardization of experimental approaches. However, the file format and data structure used in umIT, chosen to optimize processing efficiency, may present challenges for data sharing. That said, there is currently no universally accepted standard format in the field of wide-field optical imaging. As such, any format we might have adopted could face similar limitations. Data standardization remains a broader challenge across the field, and we are committed to continuing the development of the future versions toolbox to improve data accessibility and sharing with nonumIT users.

In conclusion, we believe that sharing umIT, an open-source MATLAB toolbox, will open opportunities for many research teams to more efficiently and consistently exploit their mesoscopic imaging datasets across laboratories. Although this toolbox offers a wide range of functionalities, it was also designed to enable users with little to no experience in data analysis to explore their datasets independently, without relying on programmers or needing to invest significant time in learning to code. This will also contribute to the advancement of mesoscopic imaging, benefiting biomedical and preclinical research as well as fundamental studies aimed at understanding the mechanisms of brain plasticity, development, cognition, perception, and motor functions.

## Data Availability

Source code is available on GitHub: https://github.com/LabeoTech/Umit The data used to demonstrate the functionality of this toolbox are publicly available on The Federated Research Data Repository (FRDR) at DOI: 10.20383/103.01148. They were obtained from experiments conducted for other purposes but not previously published. All experimental procedures were conducted in accordance with the Australian Code for the Care and Use of Animals for Scientific Purposes and the Australian Code for the Responsible Conduct of Research and were approved by the Animal Ethics Committee of the University of Queensland.
